# Animal-Assisted Intervention Improves Pain Perception in Polymedicated Geriatric Patients with Chronic Joint Pain: A Clinical Trial

**DOI:** 10.3390/ijerph16162843

**Published:** 2019-08-09

**Authors:** Maylos Rodrigo-Claverol, Carles Casanova-Gonzalvo, Belén Malla-Clua, Esther Rodrigo-Claverol, Júlia Jové-Naval, Marta Ortega-Bravo

**Affiliations:** 1 Primary Health Care Center Bordeta-Magraners, Catalan Institute of Health, 25001 Lleida, Spain; 2 Nursing and Physiotherapy Faculty, University of Lleida, 25198 Lleida, Spain; 3 Primary Health Care Center Primer de Maig, Catalan Institute of Health, 25003 Lleida, Spain; 4 Research Support Unit Lleida, Fundació Institut Universitari per a la recerca a l’Atenció Primària de Salut Jordi Gol i Gurina (IDIAPJGol), 25007 Barcelona, Spain

**Keywords:** animal-assisted therapy, chronic pain, elderly, primary health care

## Abstract

Chronic joint pain is associated to an increase in the consumption of medication and decrease in life quality in elderly people, which requires developing non-pharmacological treatments. The aim of this study was to evaluate the effectivity of a group intervention, based on animal-assisted therapy and applied to elderly people with chronic joint pain and polymedication, regarding the decrease of chronic pain, use of analgesics and improvement of life quality. A randomized controlled trial, two arms and open-label was conducted in a Primary Health Center. Twelve weekly sessions of kinesitherapy; in the EG, these exercises were performed with the additional assistance of the therapy dog. A total of 52 participants (22 Control Group (CG), 30 EG), average age 77.50 (±7.3), women 90.4%. A significant reduction on post-intervention values of pain β = −0.67(−1.27, −0.08), *p* = 0.03 and pain induced insomnia β = −0.53(−1.01, −0.05), *p* = 0.03 was found in EG for increasing baseline values. Animal-assisted therapy leads to an additional reduction in the perception of pain and pain induced insomnia in individuals with higher baseline severity. The presence of the dog improves the attachment to intervention and the satisfaction of the participants.

## 1. Introduction

Longevity is a characteristic of more developed societies, and increased survival leads to an increase in population ageing. Demographic forecasts predict that by 2030 more than 24% of the European population will be aged 65 or older [1], and therefore health needs are likely to continue to rise. In recent years, social and health policies targeting the elderly have undergone a major change in direction away from a fundamentally healthcare-focused dynamic, in which aging is considered to be a phenomenon defined by inactivity, disability, dependence and death. Currently, the World Health Organization (WHO) defines Active Aging as “the process of optimizing individuals’ opportunities for health, participation and safety in order to improve their quality of life as they age” [2]. Between 50% and 80% of the population over 65 years of age suffers from chronic pain [3], mainly in joints such as the shoulder, legs and feet [4]. Joint problems (arthrosis, arthritis or rheumatism) are a very common cause of chronic pain, and articular pathology is the most common cause of chronic disease in women, and the third most common in men [5]. In this population, the problem of pain is exacerbated by pluripathology and polymedication, and it is not always possible to resort to the usual drugs to alleviate it [6].

Pain affects mood, sleep patterns, and physical and social functioning, and deteriorates quality of life [7], and could be improved with effective control [8]. Chronic pain is multifactorial problem that should ideally be addressed from three main perspectives: psychological, physical and pharmacological [9]. Adding non-pharmacological interventions to pain treatment is widely recommended in the literature by both international quality control agencies [10], as well as by several clinical practice guidelines [11,12].

Primary health care has the important responsibility of taking care of elderly individuals who are frail and at risk of deteriorating health status and/or dependence. This healthcare sector accounts for most health promotion and education activities. The Education for Health initiative is intended not only to transmit information, but especially to increase the motivation, personal skills, and self-esteem necessary for individuals to adopt measures to improve their health [1]. One approach that could be used to facilitate and motivate this change in attitude in patients is the use of dogs in therapy [13].

The International Association of Human-Animal Organizations (IAHAIO) [14] defines animal-assisted interventions (AAI) as structured and targeted interventions that intentionally incorporate animals into social, health, and education contexts to achieve therapeutic and educational benefits and to improve health and well-being. Within AAI, animal-assisted therapy (AAT), as a non-pharmacological intervention, is a therapeutic modality directed by a health professional, in which the animal is used as an element that motivates and facilitates the therapy [15]. AAI techniques are based on the human-animal relationship and the bond that is generated. An important mediator of the relationship between the different effects of this human-animal interaction is oxytocin, a hormone that regulates various physiological, psychological and behavioural functions. Several studies have shown that interacting with animals can increase oxytocin levels in humans [16,17,18], and that this oxytocin release explains the observed decrease in stress and anxiety in response to contact with animals [19]. Another study reported lower levels of cortisol (indicating reduced stress) in the presence of a therapy dog [20]. In their bibliographic review, Jones et al. [21] highlight increased social interaction and psychological well-being as one of the benefits of human-animal interactions.

Various studies cite several benefits of AAT in elderly people with cognitive impairment, including: improved physical activity and depressive symptoms [22]; greater ability to reach the person at the cognitive level [23]; improvement in behavioural disorders associated with dementia [24,25]; reduced agitation and improved quality of social interactions [26,27]. Animals are very useful as a communication link during therapy sessions [28], and this has been found to have a positive effect on communication and coping ability [29]. Researchers at the Department of Medicine of the Mayo Clinic [30,31] have found that, in the hospital context, AAT is an effective therapy for patients of all ages and with various health problems. However, there is a need for evidence from the primary healthcare context. Other authors have studied the effect of AAT on the perception of pain in hospitalized [32] and postoperative children [33], and have found a significantly lower perception of pain among those receiving AAT. Studies in adults also reported improved perception of pain immediately after a hip or knee prosthesis operation [34], and among out-patients in pain units [35,36]. A randomized controlled trial found a significant reduction in pain and disability in the AAT group, and an increase in quality of life [37]. The results of this study justify the need to carry out more research on chronic pain management using this community-based approach. The most widely prescribed drugs in people over the age of 65 are those used to treat arterial hypertension and joint pathology [38]. In the context of a Rehabilitation Center, Lust et al. [39], observed a decrease in the use of analgesics in both younger and older adult patients undergoing AAT. AAT improves the patient’s attitude to and compliance with prescribed treatments, both pharmacological and lifestyle. Treatment compliance is the key to success for any intervention, and we consider that this could be improved using AAT [40].

Despite the promising results on AAI described above, there has been some criticism of the methodology used in these studies [41]. The most important limitations are considered to be the sample size, the lack of randomisation, and the absence of control groups [42]. There is a need to develop non-pharmacological methods and treatments to support a comprehensive approach to the geriatric population, and studies have shown that AAI can provide benefits for quality of life [26,27]. More studies are needed to better define fields of action and programs for the therapeutic use of animals, and to increase their use in medicine as a promising and complementary way to improve patients’ functional autonomy and quality of life [13].

The main objective of this study was to evaluate the effectiveness of a group intervention based on AAT in a geriatric population with chronic joint pain and poly-medication. We evaluated the effect of this intervention in terms of its effects on chronic pain, use of medication, and quality of life.

## 2. Materials and Methods 

### 2.1. Design and Participants

**Figure 1:**
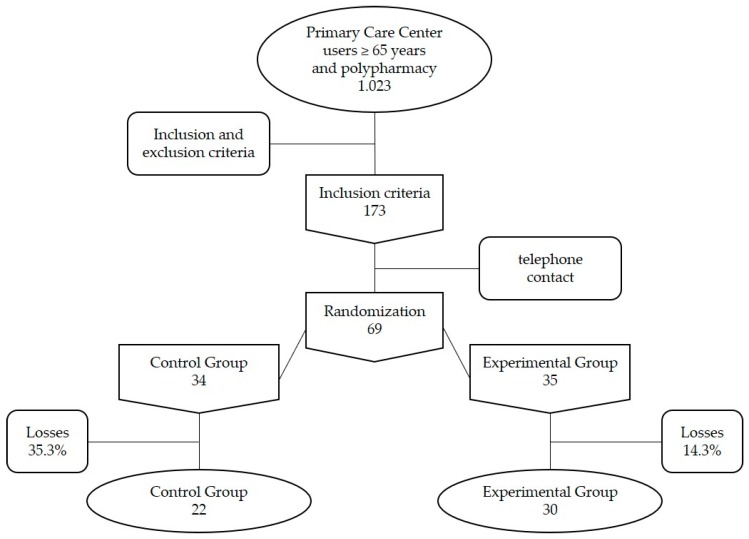
Flow diagram documenting participants included in the Clinical Trial.

We performed a single-center, two-arm randomized, controlled, open-label clinical trial. The study sample included non-institutionalized geriatric patients from an urban area who were assigned and attended by the primary healthcare clinic, and who were undergoing polymedication according to the medication plan in their computerized medical history (*n* = 1023). Of the individuals who met inclusion criteria (see below, *n* = 173), we invited, by telephone, a randomly sample (half of the individuals) to participate in the study. Those who agreed to participate were randomized by order of inclusion, one to each treatment group, until we achieved a study sample size of *n* = 69. Inclusion criteria: ≥65 years, with a diagnosis of chronic benign joint pain and polypharmacy (>5 drugs or active ingredients, of which 2 or more had been prescribed for pain). Exclusion criteria: severe cognitive deterioration (GDS > 5); allergy to or fear of animals, about which the patient was asked directly in the initial interview. After applying these criteria, the Clinical Trial included 69 participants, assigned randomly to the Control Group (CG, *n* = 34) and Experimental Group (EG, *n* = 35). The rate of dropout was 35.3% in the CG and 14.3% in the EG, which represented a borderline statistically significant difference between groups. The reasons for abandonment were incompatibility with the participant’s schedule (2 in CG, 1 in EG), illness (2 in CG, 2 in EG) and not wanting to continue participating in the intervention (8 in CG, 2 in EG). The Clinical Trial concluded with 52 participants, 22 in the CG and 30 in the EG (Figure 1).

### 2.2. Measurements

The main efficacy variable was the result of the WOMAC (Western Ontario and McMaster (WOMAC) Universities Osteoarthritis Index) psychometric questionnaire, which evaluates chronic pain, as detailed below. Secondary efficacy variables were the results of other psychometric measures: Lattinen, EuroQoL, the Health Assessment Questionnaire, and perceived pain as measured using Visual Analog Scale (VAS) [43].

#### 2.2.1. Response Variables

WOMAC Questionnaire for Arthrosis [44]: pain (0–20), stiffness (0–8), functional capacity (0–68). This questionnaire was administered before and after the intervention.

The Lattinen test [45] assesses pain and any incapacity caused by pain, as well as its frequency and intensity, the amount of painkillers taken, and whether sleep is disturbed. This questionnaire was administered before and after the intervention.

The EuroQoL Health Questionnaire (EQ-5D) [46] is a generic self-administered instrument for measuring quality of life related to health. This questionnaire was administered before and after the intervention.

The Health Assessment Questionnaire (HAQ—Spanish version) [47] is a self-administered questionnaire that assesses one’s ability to perform day-to-day activities, as well as functional capacity. This questionnaire was administered before and after the intervention.

The visual analog scale (VAS) [43] is a subjective assessment of pain that was administered before and after the intervention.

A Satisfaction Survey administered at the end of the intervention (Likert scale, 0–3) included the following questions: Did you like how the activity was conducted? Do you think these exercises could be useful for you? Would you recommend this experience to a relative and/or friend? Do you think the time-schedule and duration of the sessions are appropriate?

#### 2.2.2. Control Variables

Age (years) at the time of inclusion; sex (male/female); participant has a dog at home (yes/no); participant lives alone (yes/no). Yesavage Test (5-item version) [48], which is used to screen for depression in people over 65 years of age. This questionnaire was administered before and after the intervention.

### 2.3. General Procedures

The CG and EG underwent a therapeutic intervention based on sessions of kinesitherapy, which is defined as a set of “therapeutic procedures that use movement for the treatment and prevention of diseases of the locomotive apparatus” [49]. The experimental group also underwent AAT. We conducted a total of 12 weekly sessions of 60 min each with 10 participants. The sessions were held in the primary care center, and had specific objectives agreed in advance by the research team. The sessions had the following schedule: Session 1, lower extremities in sitting position; Session 2, upper extremities in sitting position; Session 3, cervical spine in sitting position; Session 4, dorsal rachis in sitting position; Session 5, lumbar rachis in sitting position; Session 6, Static Standing and Upper Extremities; Session 7, Static Standing; Sessions 8, 9, 10 and 11, Dynamic Standing; and Session 12, Safety Reinforcement. 

All sessions consisted of two parts. In the first part, which was the same in both the CG and EG, a series of therapeutic exercises were described, and participants were shown how to perform them correctly, so that they could do them at home during the week. In the second part, these exercises were performed in the group room with the support of various pieces of equipment (balls, spades, cones, hoops, ribbons, balloons, mats). In the EG, these exercises were performed with the additional assistance of the Therapy Dog. The therapy dog participation and the main exercises performed on each session are specified in Supplementary Table S1. All participants in each group were given a support sheet with explanations, to allow them to repeat the exercises at home.

### 2.4. Human and Animal Resources

The sessions were facilitated by a primary care nurse, a physiotherapist, and a family doctor with technical training in AAT. A nurse administered the questionnaires prior to and after the intervention. The intervention included three therapy dogs, which were selected on the basis of having a suitable character, aptitude and training that enriched the sessions. Specifically, we used a 4-year-old male Golden Retriever, and two 3-year-old female Cavalier King Charles. These dogs belong to the Ilerkan Association (www.ilerkan.com), which is a non-profit association dedicated to AAI.

### 2.5. Statistical Analysis

First, we analysed missing values in the psychometric evaluations performed throughout the trial (12 evaluations of the VAS scale), using the Fisher Test to test for differences in the number of missing values between the control and experimental groups, and the Mann-Whitney Test to compare the average number of sessions attended throughout the trial. Second, we evaluated the clinical heterogeneity of the patients assigned randomly to each group in terms of their age and sex, whether they lived alone (yes/no), and whether they had a pet dog, using the Mann-Whitney or Fisher tests depending on whether the variables were quantitative or qualitative, respectively. Similarly, we evaluated differences in the variables of interest (Womac, Lattinnen, EuroQoL, Health Assessment Questionnaire and depression) at baseline, i.e., at the beginning of the clinical trial. Third, to assess the efficacy of the intervention, we performed an analysis of covariance (ANCOVA) on all the outcome variables controlling for the respective baseline scores. For this purpose we generated a linear regression for each variable, considering the post intervention scores as the response, the group as the predictor variable and the respective baseline score as a covariate. We also considered the interaction between the group and the baseline score. All the variables were mean centered. The regression coefficients, the corresponding 95% CI and the statistical significance were estimated. All the statistical analyses were carried out using the open and free program R (www.r-project.org), and setting the level of significance to 5% (α = 0.05).

### 2.6. Ethical Considerations

The patients’ personal data were treated confidentially and were used solely for this research, in line with Spanish Organic Law 15/99 on Personal Data Protection (LOPD). This study was certified by the Clinical Research Ethics Committee (CEIC) of IDIAP Jordi Gol (code P14/027). We adhered to a protocol on animal welfare and the prevention of zoonoses, and the study was covered by civil liability insurance for dogs working in therapy.

## 3. Results

### 3.1. Description of Patients Included, Analysis of the Control Variables and Efficacy Variables at Baseline (before the Intervention)

A total of 69 patients were included, 34 in the control group and 35 in the experimental group. The dropout rate was 35.3% in the CG and 14.3% in the EG (Figure 1), which was not a statistically significant differences (*p* = 0.08, chi-squared test for independence). The final sample included 52 patients, of whom 90.4% were women, which is consistent with other pain studies that also show greater participation by women [49]. Table 1 describes the control variables, with an average age of 77.5 ± 7.3 years in the CG and 74.2 ± 7.1 years in the EG (*p* > 0.05). The percentage of women was 86.4% in the CG and 93.3% in the EG. The percentage of patients living alone was 27.3% and 30%, respectively, and that of patients living with pets was 27.3% and 33.3% (no statistically significant differences; Table 1). There was no statistically significant difference between the groups in terms of mean weight, height, body mass index, and Charlson comorbidity index (Table 1).

Before the intervention (Table 2), we observed no significant difference in the main response variable (WOMAC) or in any other variable (Lattinen, EuroQoL, HAQ, VAS), except for the stiffness dimension of the WOMAC questionnaire, which showed slightly higher values in the CG than in the EG (mean 3.8 ± 1.1 and 2.9 ± 1.3, respectively; *p* = 0.02). In contrast, we found no significant difference in depression between the groups, as evaluated using the Yesavage questionnaire.

### 3.2. Evaluation of the Effectiveness of the Intervention via the Main Variable

**Figure 2:**
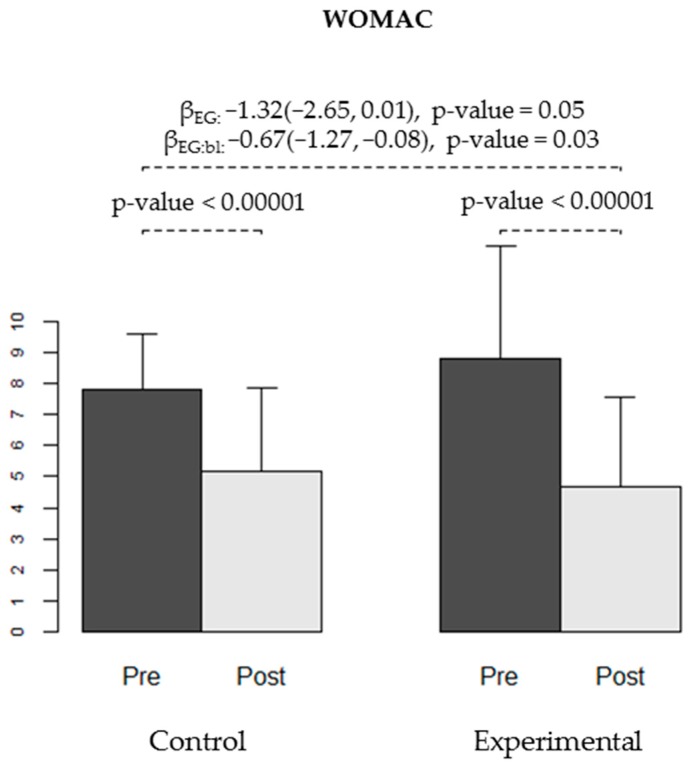
Pain level measured using the Western Ontario and McMaster Universities Osteoarthritis Index (WOMAC) test at baseline (pre-) and at the end of the trial (post-intervention) in both groups (CG and EG). The bars show the average level of pain observed in each group for each time-point, and the error bars represent the standard deviation. The *p*-values for the pain reduction obtained in each group are shown. Regression coefficients for experimental group (βEG), for the interaction between group and baseline score (βEG:bl) and respective 95% confidence intervals and *p*-values are displayed.

**Figure 3:**
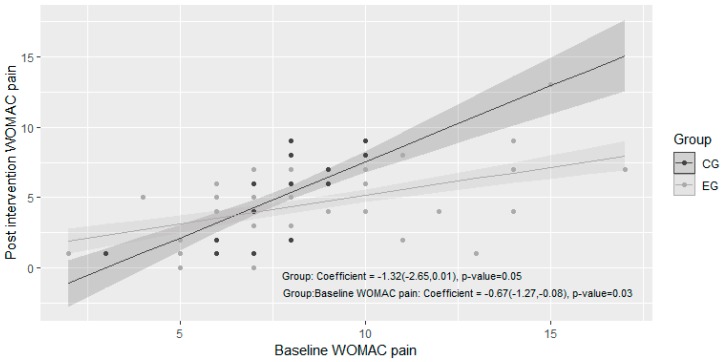
Linear regression for the response variable “post intervention WOMAC pain score” considering the group as the predictor variable and the baseline Western Ontario and McMaster Universities Osteoarthritis (WOMAC) pain score as a covariate. The points represent the real values of each individual and the lines represent the fitted model, with a grey shade representing the 95% confidence intervals for the predictions. The regression coefficients regarding the group effect are shown, with the corresponding 95% CI and *p*-value.

We observed a statistically significant decrease in the WOMAC pain scale after the intervention in both the CG and EG (*p* < 0.00001, Table 3, Figure 2). When analysing the effect of AAT on post intervention WOMAC pain scale, we observed a reduction on post intervention pain of 1.32 points. This difference was borderline significant (*p* = 0.05). We also observed an effect of 0.67 points of reduction on the interaction between EG and baseline WOMAC pain (95% CI −1.27, −0.08, *p =* 0.03), indicating that for each increment of 1 point on baseline WOMAC pain, the EG achieved a reduction on post intervention WOMAC pain of 0.67 points compared to the CG (Table 4, Figure 3). As a whole, these results indicate that the intervention is significantly more effective in reducing pain when it is assisted by therapy dogs, with higher effects on individuals with more painful symptoms.

Effect of the animal-assisted therapy, calculated with a linear regression for each variable, considering the post intervention scores as the response, the group as the predictor variable and the respective baseline score as a covariate. Regression coefficients for experimental group (β_EG_), for the interaction between group and baseline score (β_EG:bl_), 95% confidence intervals and *p*-values are displayed. WOMAC: Western Ontario and McMaster Universities Osteoarthritis Index; HAQ: Health Assessment Questionnaire; VAS: Visual Analog Scale.

### 3.3. Evaluation of the Effectiveness of the Intervention via the Secondary Variables

When analysing the secondary variables, we observed a significant effect of −0.53 on the interaction between the group and baseline pain induced insomnia (95% CI −1.01, −0.05, *p* = 0.02). Regarding analgesics consumption, pain frequency and incapacity, while the EG group showed a significant difference in pre-post values and the CG did not, there was no significant main effect of group on post-treatment values controlling for baseline use, and no interaction between group assignment and baseline scores (Table 3 and Table 4).

**Figure 4:**
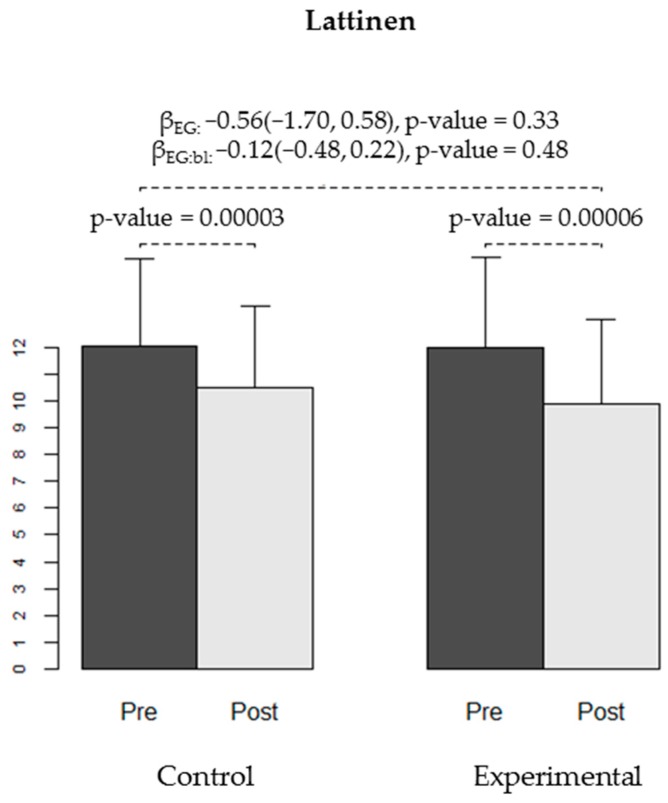
Pain level measured using the Lattinen test at baseline (pre-) and at the end of the trial (post-intervention) in both groups (CG and EG). The bars show the average level of pain observed in each group at each time-point, and the error bars represent the standard deviation. The *p*-values for pain reduction obtained in each group are shown. Regression coefficients for experimental group (βEG), for the interaction between group and baseline score (βEG:bl) and respective 95% confidence intervals and *p*-values are displayed.

**Figure 5:**
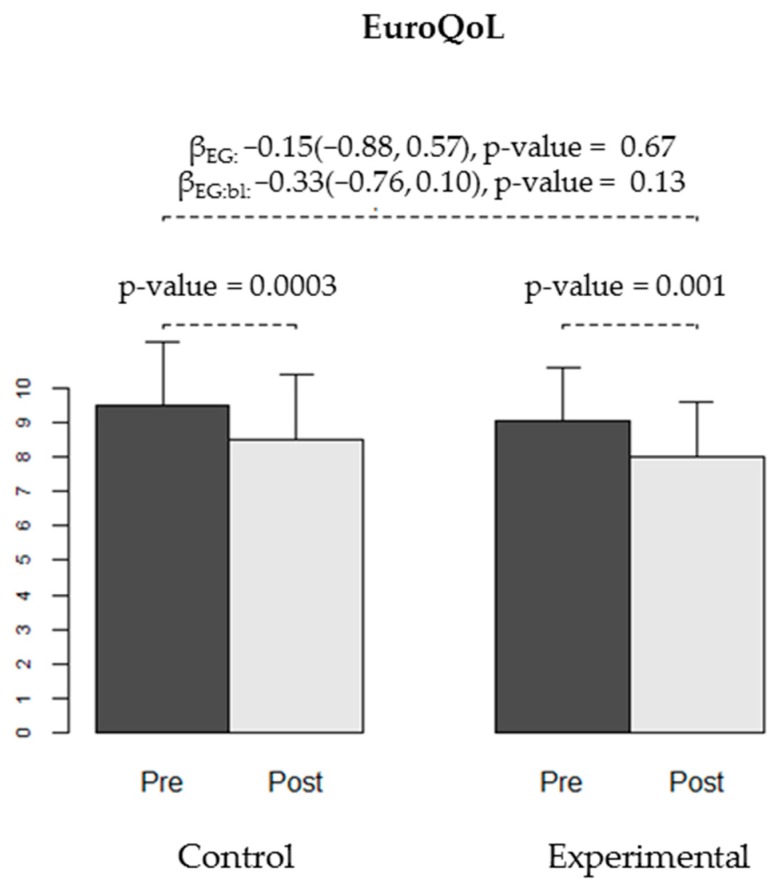
Quality of Life measured using the EuroQoL test at baseline (pre-) and at the end of the trial (post-intervention) in both groups (CG and EG). The bars show the average quality of life observed in each group at each time-point, and the error bars represent the standard deviation. The *p*-values for improvement in quality of life obtained in each group are shown. Regression coefficients for experimental group (βEG), for the interaction between group and baseline score (βEG:bl) and respective 95% confidence intervals and *p*-values are displayed.

**Figure 6:**
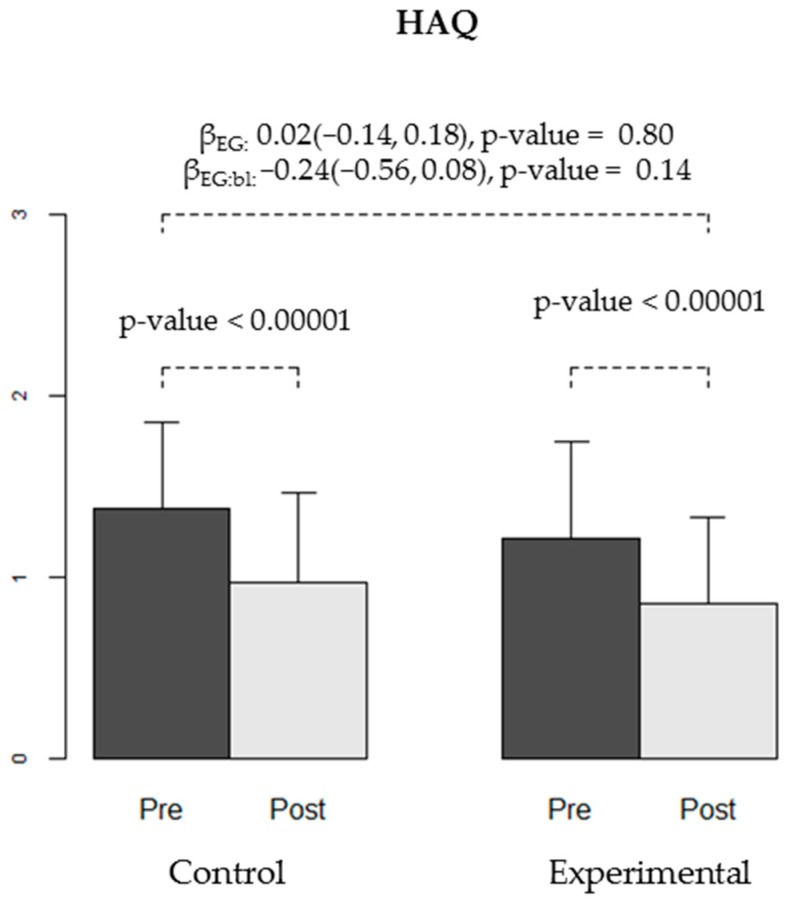
Quality of life measured using the Health Assessment Questionnaire (HAQ) test at baseline (pre-) and at the end of the trial (post-intervention) in both groups (CG and EG). The bars show the average quality of life observed in each group at each time-point, and the error bars represent the standard deviation. The *p*-values for improvement in quality of life obtained in each group are shown. Regression coefficients for experimental group (βEG), for the interaction between group and baseline score (βEG:bl) and respective 95% confidence intervals and *p*-values are displayed.

**Figure 7:**
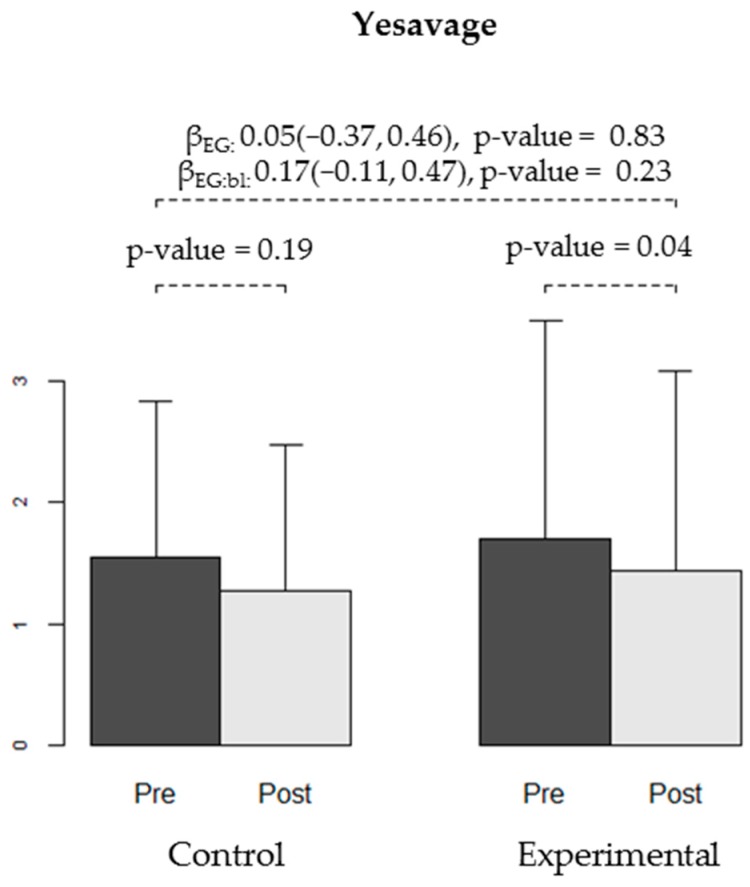
Depressive symptoms measured using the Yesavage test at baseline (pre-) and at the end of the trial (post-intervention) in both groups (CG and EG). The bars show the average level of depressive symptoms observed in each group at each time-point, and the error bars represent the standard deviation. The *p*-values for the decrease in depressive symptoms reduction obtained in each group are shown. Regression coefficients for experimental group (βEG), for the interaction between group and baseline score (βEG:bl) and respective 95% confidence intervals and *p*-values are displayed.

We found no differences in the effectiveness of the intervention between the CG and EG for any of the secondary variables: WOMAC stiffness and functional capacity, VAS, Lattinen (in general or for any of the sub-dimensions), EuroQoL quality of life, HAQ, or depression (Yesavage) (Table 4, Figure 4, Figure 5, Figure 6 and Figure 7).

### 3.4. Evaluation of the Effectiveness of the Intervention Based on the Level of Pain Measured in Each Session Using the VAS Scale

We used the VAS scale to measure perceived pain levels before each session, and assessed whether these pain levels were lower after the session. In Sessions 11 and 12, the observed decrease in pain in the EG (1.16 ± 1.43 and 0.8 ± 1.17, respectively) was significantly greater (*p* = 0.04, *p* = 0.02, Table 5) than that observed in the CG (0.37 ± 0.84 and −0.2 ± 1.55). A similar pattern was observed in Session 1, with borderline statistical significance.

### 3.5. Evaluation of Patient Satisfaction

There was no statistically significant difference in the average level of satisfaction reported by subjects in the CG compared to those in the EG (Table 6). However, a significantly (Chi-square test, *p* = 0.0002) greater number of participants in the EG provided a written response in the comments section of the satisfaction survey, *n* = 25, compared to just 6 participants in the CG.

## 4. Discussion

The objective of this study was to evaluate the effect of an AAT-based health education intervention in elderly individuals suffering from chronic joint pain. We also assessed the effect of the intervention on the patients’ consumption of analgesics and quality of life.

Our results show a decrease in the primary variable, WOMAC-pain, in both groups, with a significantly higher effect of the AAT on individuals with more painful symptoms. These results suggest that AAT might allow a better performance on the sessions, either by distracting the participants or by promoting a higher engagement with the exercises, and therefore reducing the subjective perception of pain during the session [50]. This might become especially relevant on cases with higher pain. In the Lattinen Test, the overall result reflects a significant improvement in both the CG and the EG, taken separately. Analysing the different dimensions of this test, we found a significant decrease in pain induced insomnia in the EG compared to the CG for participants with higher baseline insomnia. We also observed a significant decrease in pain intensity in each group, whereas the frequency of pain and the associated disability only decreased significantly in the EG. In a clinical trial of 72 patients undergoing total hip arthroplasty, Harper et al. [34] assessed the effect of AAT on the perception of pain using the VAS scale, and observed a significantly greater decrease in the perception of pain and ability to manage pain [34]. In our study, we administered the VAS test at the beginning and end of each session. In the first session, there was a borderline significant difference in pain reduction between groups (*p* = 0.054), possibly due to the novelty of the presence of the dog in the therapeutic environment, while in sessions 11 and 12, this decrease in pain was significantly greater in the EG (*p* = 0.04 and *p* = 0.02, respectively). This improvement can be attributed to the more dynamic nature of the sessions, and the more active participation of the patients in the exercises, which favours a more direct patient-animal interaction.

Pain is a complex sensory and emotional experience that includes perceptions, emotions and behaviours, and may become disabling [50,51]. Therapies that have an emotional, sensorial, and affective influence on the components of pain perception can break the cycle of chronic pain [52]. Since the perception of pain is highly subjective, we decided to evaluate pain using three different measures. Regarding the pain evaluation in our study, the baseline result using the VAS scale would be equivalent to moderate-severe pain according to the Lattinen test, and quite painful according to the WOMAC questionnaire. Regarding pain intensity, Gordillo et al. obtained similar results to ours in that most patients with chronic pain report that they had moderate to severe pain [53]. Another recent study suggests that dog ownership provides relief and support for chronic lower back pain [54]. In a study of 382 outpatients of a Pain Unit, Marcus et al. [35] showed that the presence of therapy dogs in the waiting room could significantly reduce pain and emotional stress, and improve emotions and feelings of well-being among the family, companions, and health professionals. In a later study, these authors also observed that brief visits of therapy dogs provided a valuable complementary therapy for outpatients with fibromyalgia, resulting in a significant reduction in pain and anguish [55]. Research into pain reduction and pain-related symptoms has shown that patients who receive therapy dog visits show a subsequent decrease in catecholamines and stress hormones such as adrenaline and norepinephrine, and an increase in endorphins and in their pain threshold [16,17,36,56,57,58,59].

In another controlled clinical trial carried out in Italy, Calcaterra et al. [60] assessed the impact of an AAT program on children’s response to stress and pain immediately post-surgery. They found that the AAT facilitated rapid recovery of activity after anaesthesia and lower perception of pain, and provoked emotional prefrontal responses [60]. Similarly, a clinical trial among hospitalized children in the US found a four-fold greater decrease in pain among those receiving AAT, and the authors noted that this effect was comparable to that of a dose of paracetamol in adults [32]. In our study, we observed a statistically significant decrease in intake of analgesics, as measured by the Lattinen test, in the EG, but not in the CG. However, we found no statistically significant effect of group on post-treatment analgesic use controlling for baseline use. Miller and Ingram [61] were able to reduce the use of painkillers in surgical patients undergoing AAT, while Geisler observed that patients participating in AAT programs reduced their intake of psychotropic medications, and thereby reduced their health costs [62].

Chronic pain has a multidimensional effect on sufferers’ quality of life [63], which could be improved by minimizing pain [64]. The baseline results from our quality of life questionnaires (EuroQoL and HAQ) indicate that patients experienced pain in performing daily life activities, and that this was significantly improved in both study groups after the intervention. This result is consistent with those of recent research in elderly institutionalized people [26,65], and in adults with chronic schizophrenia [66], that also noted a similar effect of intervention in both CG and EG. Fleishman et al. [67] evaluated the effect of therapy dog visits on perceived quality of life of cancer patients during radiotherapy and chemotherapy sessions, and reported a significant improvement in emotional well-being [67]. Other authors highlight the importance of AAT as a positive feedback mechanism between the patient, the animal, and the health professional that can improve many symptoms as well as quality of life [68]. Other studies indicate that AAI can improve quality of life in the geriatric population [23,24,25,26,27], such as in one study where nursing registries showed that the 83 participants experienced joy and well-being during and after the therapy sessions with dogs [69]. Positive emotions are known to increase the efficacy of treatments, with both physiological and psychological benefits [70], and to improve one’s way of thinking [71,72], well-being, quality of life [73,74], and resilience [75,76]. During the sessions in our study, we observed that therapy dog was a topic of conversation and turned out to be the center of attention in the EG, allowing us to generate a more relaxed atmosphere and greater group cohesion.

Another interesting observation is the high rate of follow-up and commitment to the AAT in the EG, as reflected in attendance at the sessions. This leads us to believe that the novelty of introducing pets into patients’ usual treatment makes this type of intervention suitable for helping to focus their attention and work more effectively in areas of interest. The aim of AAT is never to be independent or to replace other interventions, but as a complement to traditional interventions [66]. The drop-out rate in our study was 24.6% overall, but almost twice as high in the CG as in the EG. It is important to remark that the drop-out rate for personal reasons (not wanting to continue participating in the intervention) was 4 times higher in the CG suggesting that AAT contributes to a better adherence to the therapy. In comparison, Nordgren et al. [26] observed a higher dropout rate than in our study, at 50%. The participants in our study expressed their satisfaction and willingness to repeat the experience, a similar finding to those of other studies where participants also expressed a desire for future visits with therapy dogs [55]. In our study, 83.3% of participants in the EG provided a positive written comment in the observations section of the satisfaction survey, compared to only 27.3% in the CG (*p* = 0.0002).

Various studies have evaluated the effects of AAT, and conclude that it provides class IIa–IIb evidence, which is considered acceptable to recommend AAT for optimizing the work of health professionals [77]. Previous studies suggest that the presence of an animal facilitates the relationship between the therapist and the patient [21,78,79], while another showed that the interaction between the patient, animal and health professional improves communication, reduces disease symptoms and improves quality of life [80]. We found that the therapy dog favours a more pleasant memory of the activity, facilitates learning, and increases participants’ ability to perform the prescribed exercises. It also improves adherence to the intervention, as individuals in the EG went to more sessions than those in the CG, which is key to the success of any group intervention.

One of the limitations of this study is the final sample size, which may limit our statistical power. Increasing the sample size, may allow us to observe a significant effect, not only for the main variable, but also for some of the secondary variables. Nonetheless, our results highlight the marked, and statistically significant, effect of AAT as a complementary therapy to achieve greater pain reduction among geriatric patients with chronic joint pain. Another limitation is the open-label design of the study, which could not be double blinded because of the nature of the intervention. To minimize this limitation, the questionnaires were self-administered. The implicit subjectivity of aspects such as pain perception and quality of life could be another limitation, which we attempted to minimize by using two different questionnaires for each aspect.

Our study was carried out in a primary care setting, and responds to the need to improve pain management and provide an innovative and non-pharmacological approach, in this case using health education through AAT to create healthier lifestyle habits by enhancing physical exercise in patients with chronic pain. The results of our study highlight the value of future applications of AAI to complement health education and promotion of active aging in Primary and Community Care. This study provides evidence for AAI and postulates a need for future research to evaluate the long-term effects of AAT and to analyse the cost-effectiveness of the intervention.

## 5. Conclusions

AAT as a complementary therapy facilitated an additional reduction in the perception of pain and pain induced insomnia in individuals with higher baseline severity. Quality of life improved in both groups. In addition, AAT increased adhesion to the intervention and reduced the drop-out rate. AAT contributes to the development of methods and non-pharmacological treatments, which have become an important tool for managing chronic pain as a complement to the pharmacological treatment.

## Figures and Tables

**Table 1:** Differences between the control and experimental group in terms of the baseline clinical and anthropometric variables.

Variables	Groups			
	All Patients *n* = 52 (100%)	Control *n* = 22 (42.31%)	Experimental *n* = 30 (57.69%)	*p*-Value
Age	75.60 (7.29)	77.50 (7.30)	74.20 (7.08)	0.11
Gender				0.64
man	5 (9.62%)	3 (13.64%)	2 (6.67%)	
woman	47 (90.38%)	19 (86.36%)	28 (93.33%)	
Weight	70.89 (12.5)	72.25 (13.34)	69.03 (11.29)	0.23
Height	153.76 (8.13)	152.71 (6.63)	155.2 (9.8)	0.63
Body Mass Index	29.96 (4.91)	30.85 (5.38)	28.74 (3.98)	0.09
Charlson Index	1.56 (1.97)	1.5 (1.5)	1.64 (2.52)	0.45
Number of sessions held	9.35 (1.80)	8.82 (1.65)	9.73 (1.84)	0.06
Live alone				1
live alone	15 (28.85%)	6 (27.27%)	9 (30%)	
no live alone	37 (71.15%)	16 (72.73%)	21 (70%)	
Pet				0.76
no pet	36 (69.23%)	16 (72.73%)	20 (66.67%)	
live with pet	16 (30.77%)	6 (27.27%)	10 (33.33%)	

Average (and standard deviation) or absolute frequency (and percentage) shown by quantitative and qualitative variables, respectively. Differences were assessed using the Mann-Whitney test for quantitative variables and the Fisher test for qualitative variables, and by calculating the corresponding *p*-value.

**Table 2:** Differences between the control and experimental group in terms of the efficacy variables at baseline (pre-intervention).

Basal Response Variables	Groups			
	All Patients *n* = 52 (100%)	Control *n* = 22 (42.31%)	Experimental *n* = 30 (57.69%)	*p*-Value
WOMAC				
General	8.40 (3.03)	7.82 (1.82)	8.83 (3.65)	0.57
Stiffness	3.27 (1.30)	3.77 (1.11)	2.90 (1.32)	0.02
Functional capacity	27.67 (8.53)	27.32 (7.21)	27.93 (9.49)	0.91
Lattinen				
General	12.06 (3.30)	12.09 (3.28)	12.03 (3.38)	0.72
Intensity	2.50 (0.90)	2.59 (0.96)	2.43 (0.86)	0.7
Frequency	2.83 (0.90)	2.77 (0.92)	2.87 (0.90)	0.79
Analgesics	2.12 (0.83)	1.91 (0.81)	2.27 (0.83)	0.11
lncapacity	1.94 (0.64)	2.00 (0.62)	1.90 (0.66)	0.58
Pain induced insomnia	2.46 (1.31)	2.68 (1.25)	2.30 (1.34)	0.27
EuroQoL	9.25 (1.68)	9.50 (1.85)	9.07 (1.55)	0.4
HAQ	1.28 (0.51)	1.38 (0.47)	1.21 (0.54)	0.14
Yesavage depression	1.63 (1.60)	1.55 (1.30)	1.70 (1.80)	0.93
Baseline Session 1 VAS	4.98 (2.54)	4.89 (2.95)	5.04 (2.31)	0.94

Average (and standard deviation) shown. Differences were assessed using the Mann-Whitney test and by calculating the corresponding *p*-value. WOMAC: Western Ontario and McMaster Universities Osteoarthritis Index; HAQ: Health Assessment Questionnaire; VAS: Visual Analog Scale.

**Table 3:** Evaluation of the effectiveness of the intervention on the variables of interest.

Response Variables	Control Group *n* = 22 (42.31%)						Experimental Group *n* = 30 (57.69%)					
	Pre	Post	Difference (Post-Pre)	95% CI	*p*-Value		Pre	Post	Difference (Post-Pre)	95% CI	*p*-Value	
					A	B					A	B
WOMAC												
Pain	7.82 (1.82)	5.18 (2.67)	−2.64 (1.81)	(−3.44, −1.83)	<0.00001	0.00008	8.83 (3.65)	4.67 (2.9)	−4.17 (3.31)	(−5.4, −2.93)	<0.00001	<0.00001
Stiffness	3.77 (1.11)	2.5 (1.22)	−1.27 (1.35)	(−1.87, −0.67)	0.0002	0.001	2.9 (1.32)	2.3 (1.24)	−0.6 (1.16)	(−1.03, −0.17)	0.008	0.009
Functional capacity	27.32 (7.21)	19.95 (7.52)	−7.36 (5.55)	(−9.82, −4.9)	<0.00001	0.0002	27.93 (9.49)	17.13 (8.61)	−10.8 (7.1)	(−13.45, −8.15)	<0.00001	<0.00001
Lattinen												
General	12.09 (3.28)	10.5 (3.05)	−1.59 (1.74)	(−2.36, −0.82)	0.0003	0.002	12.03 (3.38)	9.9 (3.17)	−2.13 (2.49)	(−3.06, −1.2)	0.00006	0.00008
Intensity	2.59 (0.96)	1.86 (0.89)	−0.73 (0.83)	(−1.09, −0.36)	0.0005	0.002	2.43 (0.86)	1.93 (0.87)	−0.5 (0.9)	(−0.84, −0.16)	0.005	0.007
Frequency	2.77 (0.92)	2.41 (1.18)	−0.36 (0.85)	(−0.74, 0.01)	0.06	0.06	2.87 (0.9)	2.1 (0.84)	−0.77 (0.9)	(−1.1, −0.43)	0.00006	0.0004
Analgesics	1.91 (0.81)	1.86 (0.71)	−0.05 (0.58)	(−0.3, 0.21)	0.71	0.78	2.27 (0.83)	1.93 (0.83)	−0.33 (0.66)	(−0.58, −0.09)	0.01	0.02
lncapacity	2 (0.62)	1.82 (0.5)	−0.18 (0.5)	(−0.4, 0.04)	0.1	0.13	1.9 (0.66)	1.63 (0.61)	−0.27 (0.52)	(−0.46, −0.07)	0.009	0.01
Pain induced insomnia	2.68 (1.25)	2.5 (1.41)	−0.18 (0.59)	(−0.44, 0.08)	0.16	0.2	2.3 (1.34)	2.27 (1.44)	−0.03 (1.45)	(−0.57, 0.51)	0.9	0.75
EuroQoL	9.5 (1.85)	8.5 (1.9)	−1 (1.07)	(−1.47, −0.53)	0.0003	0.0008	9.07 (1.55)	8.03 (1.61)	−1.03 (1.56)	(−1.62, −0.45)	0.001	0.002
HAQ	1.38 (0.47)	0.97 (0.49)	−0.4 (0.23)	(−0.5, −0.3)	<0.00001	0.00007	1.21 (0.54)	0.85 (0.47)	−0.35 (0.34)	(−0.48, −0.23)	<0.00001	0.0001
Yesavage	1.55 (1.3)	1.27 (1.2)	−0.27 (0.94)	(−0.69, 0.14)	0.19	0.22	1.7 (1.8)	1.43 (1.65)	−0.27 (0.69)	(−0.52, −0.01)	0.04	0.049
Reduction VAS (Pre—Post)	0.24 (0.90)	0.06 (0.74)	−0.17 (1.06)	(−0.79, 0.44)	0.56	0.53	1.26 (1.63)	0.74 (1.20)	−0.52 (1.53)	(−1.15, 0.11)	0.11	0.09

Effect observed in CG and EG calculated as the difference in the mean value observed at the end of the trial (post) with respect to the corresponding value at baseline (pre-intervention). Average values observed in each group pre- and post-intervention and the mean of the effect (with the corresponding standard deviation) are shown, with the corresponding 95% CI, assessed using a Student T test (A) or a Wilcoxon (B) test for paired data. WOMAC: Western Ontario and McMaster Universities Osteoarthritis Index; HAQ: Health Assessment Questionnaire; VAS: Visual Analog Scale.

**Table 4:** Evaluation of the experimental group effect on the post intervention scores.

Response Variables	EG Effect on Post Intervention Scores					
	β_EG_	95% CI	*p*-Value	β_EG:bl_	95% CI	*p*-Value
WOMAC						
Pain	−1.32	(−2.65, 0.01)	0.05	−0.67	(−1.27, −0.08)	0.03
Stiffness	0.19	(−0.47, 0.85)	0.57	0.18	(−0.35, 0.71)	0.49
Functional capacity	−3.25	(−6.60, 0.10)	0.06	−0.12	(−0.55, 0.31)	0.60
Lattinen						
General	−0.56	(−1.70, 0.58)	0.33	−0.12	(−0.48, 0.22)	0.48
Intensity	0.15	(−0.28, 0.58)	0.49	−0.10	(−0.58, 0.39)	0.69
Frequency	−0.37	(−0.83, 0.08)	0.10	−0.46	(−0.96, 0.05)	0.08
Analgesics	−0.16	(−0.50, 0.17)	0.32	0.04	(−0.35, 0,45)	0.8
lncapacity	−0.12	(−0.38, 0.11)	0.30	0.12	(−0.28, 0.52)	0.54
Pain induced insomnia	0.07	(−0.54, 0.68)	0.82	−0.53	(−1.01, −0.05)	0.02
EuroQoL	−0.15	(−0.88, 0.57)	0.67	−0.33	(−0.76, 0.10)	0.13
HAQ	0.02	(−0.14, 0.18)	0.80	−0.24	(−0.56, 0.08)	0.14
Yesavage	0.05	(−0.37, 0.46)	0.83	0.17	(−0.11, 0.47)	0.23

**Table 5:** Evaluation of the effectiveness of the intervention using the VAS pain scale.

Session	Reduction in VAS (Pre—Post Session)		Difference in the Reduction (Experimental vs. Control)			
	Control Group *n* = 22 (42.31%)	Experimental Group *n* = 30 (57.69%)	Difference	95% CI	*p*-Value	
					A	B
Session 1	0.43 (01.03)	1.26 (1.63)	0.83	(−0.015, 1.66)	0.054	0.21
Session 2	0.68 (1.61)	1.55 (1.95)	0.87	(−0.15, 1.89)	0.09	0.044
Session 3	0.89 (1.50)	0.70 (1.36)	−0.19	(−1.05, 0.68)	0.66	0.81
Session 4	1.24 (2.54)	0.71 (1.95)	−0.53	(−2.11, 1.04)	0.49	0.48
Session 5	−0.018 (2.56)	0.69 (1.56)	0.71	(−0.72,2.15)	0.31	0.69
Session 6	0.41 (1.48)	0.87 (1.81)	0.45	(−0.56, 1.48)	0.37	0.39
Session 7	1.00 (2.14)	0.35 (1.03)	−0.65	(−2.00, 0.70)	0.33	0.36
Session 8	1.06 (1.06)	0.31 (1.25)	−0.75	(−1.54, 0.042)	0.063	0.12
Session 9	0.40 (1.05)	0.80 (1.64)	0.40	(−0.45, 1.25)	0.34	0.44
Session10	0.90 (1.11)	0.92 (1.16)	0.02	(−0.91, 0.96)	0.96	0.98
Session11	0.37 (0.84)	1.16 (1.43)	0.79	(0.06, 1.53)	0.04	0.02
Session12	−0.2 (1.55)	0.8 (1.17)	1.00	(0.15, 1.85)	0.02	0.02

Average (and standard deviation) of the observed decrease in pain for each session in both groups (CG and EG). The difference between the decreases in experimental and control groups are shown, including the corresponding 95% CI and *p*-value using the Student T test (A) and a Mann-Whitney (B) test.

**Table 6:** Differences in satisfaction between the control and experimental group as reported at the end of the trial.

Satisfaction Variables	Groups			
	All Patients *n* = 52 (100%)	Control *n* = 22 (42.31%)	Experimental *n* = 30 (57.69%)	*p*-Value
Did you like how the activity has developed?	2.90 (0.36)	2.90 (0.44)	2.90 (0.31)	0.55
Do you consider that the exercises performed can be useful?	2.75 (0.48)	2.81 (0.51)	2.70 (0.47)	0.25
Would you recommend this experience to a family member or friend?	2.84 (0.37)	2.76 (0.44)	2.90 (0.31)	0.19
Do you consider the session schedule adequate?	2.39 (0.63)	2.38 (0.59)	2.40 (0.67)	0.81
And its duration?	2.45 (0.76)	2.33 (0.80)	2.53 (0.73)	0.33

Average (and standard deviation) satisfaction, on a scale of 0 (not at all satisfied) to 3 (very satisfied). Differences were evaluated using a Mann-Whitney test, and the corresponding *p*-value.

## Supplementary Materials

The following are available online at https://www.mdpi.com/1660-4601/16/16/2843/s1. Table S1: Objectives, exercises and descriptions of therapy dog participation in each session.
