# Supplementary material for: Animal-Assisted Intervention Improves Pain Perception in Polymedicated Geriatric Patients with Chronic Joint Pain: A Clinical Trial
Source: Int J Environ Res Public Health. 2019 Aug 9;16(16):2843. doi: 10.3390/ijerph16162843 (PMC6721103; doi:10.3390/ijerph16162843)
Supplement: Supplementary file 1 [file ijerph-16-02843-s001.pdf]

Supplementary Table 1. Objectives, exercises and descriptions of therapy dog participation in each session.

SESSION	FOCUS	EXERCISES	THERAPY DOG PARTICIPATION
1	Lower extremities in sitting position	1. Toe to heel, trampling, crossing legs above knee.<br>2. Knee bending-stretching.<br>3. Ball shooting.<br>4. Passing a small hoop from the foot to the hip.	1. The therapy dog marks the shift of the participant's exercise based on specific gestures.<br>2. The therapy dog sits in front of the participant, who has to extend his leg until he/she touches the dog with his/her foot.<br>3. The therapy dog fetches the balls.<br>4. The therapy dog distributes the hoops.
2	Upper extremities in sitting position	1. Gathering a ball from the ground with one hand.<br>2. Gathering a ball from the ground with two hands.<br>3. Sitting in a circle and passing a ball to the participant sitting on his/her side.<br>4. Passing a big hoop with two hands from the feet to hip height.	1, 2. The therapy dog lies on the side of the participant. The participant has to pet the dog performing lateral flexion of the trunk while gathering the ball.<br>3. The therapy dog sits in the middle and when indicated by the physiotherapist, the ball is thrown at the dog.<br>4. The therapy dog distributes hoops and at the end, a tunnel of hoops is made so the dog can pass through it.
3	Dorsal spine in sitting position	Different exercises for working on:<br>1. Dorsal spine flexion.<br>2. Dorsal spine extension.<br><br>Between the different exercises, the participants roll one dice in order to decide which skill the dog has to perform.	1. The therapy dog lies between the legs of the participant. He/she must pet it with the back of his hand.<br>2. The therapy dog sits side-back of the participant and he/she must ask for the dog's paw<br>1, 2. The therapy dog sits in front of the participant and he/she must reach the dog with a slalom bar by flexing his/her back.<br>1, 2. The participant throws a ball to the dog with 2 hands in order to perform a dorsal spine flexion and extension.
4	Cervical rachis in sitting position	1. Rolling a small ball from the hand to the neck with the arm in different abduction degrees.<br>2. Passing a hoop from the hand to the neck with the arm in different abduction degrees.<br>3. Lateral tilt, flexion and extension cervical exercises.	1. The therapy dog is in the center of the room and participants must throw the ball directly into the dog's mouth (without it falling to the ground).<br>2. The participants put the hoops on the neck of the therapy dog that is sitting in front of them.<br>3. The therapy dog waits with the participants while performing the exercises.
5	Lumbar rachis in sitting position	1. Raising a slalom pole from the knees to different heights.<br>2. Lifting/lowering a ribbon held by two people.	1. Before starting the exercise the participants ask the therapy dog to spin around the pole.<br>2. The dog goes under or jumps over the ribbon.
6	Static Standing and Upper Extremities	1, 2. Throwing a ball following different instructions.	1. The participants play basketball with the therapy dog.<br>2. The therapy dog is located in the middle of the room. Each half of the

			group is on each side of the room and throws the balls over the dog.
7	Static Standing	1. Bouncing a ball with one or two hands.<br>2. Passing a ball from one hand to another through the lower back.<br>3. Kicking a ball following the instructions: right / left leg, sideways ...	1. The therapy dog passes between the participants while performing the exercise.<br>2. The exercise is performed with food instead of a ball for the therapy dog to surround the participants.<br>3. The therapy dog acts as a goalkeeper while the participants kick the ball.
8-11	Dynamic Standing	1, 2, 3. Exercises to work the forward, backward and sideways march.<br>4. Exercise to work height changes.<br>5. Short paths with orientation changes.	1. The therapy dog sits at other end of the room and the participants have move to put the collars to their dogs.<br>2. Slalom with legs: the participant walks with long steps and therapy dog has to walk under the participant's legs.<br>3. The therapy dog and participants together perform spinning exercises on themselves.<br>4. The participants pick up a hoop from the ground or from a height and put it on the dog's neck.<br>5. Different tracks with cones to perform zigzag and with slalom poles to pass above with the therapy dog.
12	Safety Reinforcement	1. Participants in a row are passing a ball. Participants in a row swing their arms with a slalom pole.<br>2. Working the march from sitting position.<br>3. Standing up from the floor from sitting and from lying.	1. While performing the exercise the therapy dog walks under the participants legs as a tunnel.<br>2. The participants "run" a relay race accompanied by the therapy dog.<br>3. The therapy dog unfolds a pad so that the participant can lie on the floor.
